# Factors associated with early sexual onset and delaying sex in rural middle school youth

**DOI:** 10.1111/jrh.12889

**Published:** 2024-10-07

**Authors:** Teresa M. Imburgia, Devon J. Hensel, Abby Hunt, Rebecca James, Jianjun Zhang, Michele L. Cote, Mary A. Ott

**Affiliations:** ^1^ Department of Pediatrics Indiana University School of Medicine Indianapolis Indiana USA; ^2^ Department of Epidemiology Indiana University Richard M. Fairbanks School of Public Health Indianapolis Indiana USA; ^3^ Department of Sociology Indiana University Purdue University Indianapolis Indiana USA; ^4^ Health Care Education and Training Indianapolis Indiana USA; ^5^ The Arnhold Institute for Global Health Icahn School of Medicine at Mount Sinai New York New York USA

**Keywords:** adolescent, early sexual onset, rural youth

## Abstract

**Purpose:**

Early sexual onset contributes to poor health outcomes through the life course. We use the social behavioral model to examine the behaviors and attitudes associated with early sexual onset and the intention to delay sex in middle school youth.

**Methods:**

Youth in rural communities with high rates of hepatitis C and HIV filled out a survey prior to implementation of an evidence‐based sex education program. Participants were asked if they had ever had sex and whether they planned to abstain from sex until the end of high school. We collected demographics, attitudes about abstinence, agency for sexual refusal, parent communication, sexual health knowledge, and history of system involvement. Logistic regression was utilized to examine factors associated with each outcome.

**Findings:**

Our sample included 6,799 students, 12.7 years old ± 0.9 and 50.3% female. 5.1% had ever had sex and 73.9% planned to abstain until the end of high school. Early sexual onset was associated with older age, negative attitudes toward abstinence, lower agency for sexual refusal, more frequent parent communication about sex, history of child welfare, and history of juvenile involvement. Planning to abstain until the end of high school was associated with being younger, female, positive attitudes toward abstinence, higher agency for sexual refusal, less communication with parents about sex, more communication with parents about relationships, not having a history of foster involvement, and not having a history of juvenile involvement.

**Conclusions:**

Age, agency, and parent communication were all associated with both outcomes. Our findings highlight the importance of early comprehensive, trauma‐informed sex education.

## INTRODUCTION

### Early sexual onset

Sexual behavior is common during middle to late adolescence and is considered a part of normal development.^1^, ^2^ During early adolescence, however, sexual intercourse is less common, often coerced,^3^ and is associated with poor health outcomes.^4^ The 2021 Youth Risk Behavior Survey, a set of surveys sponsored by the Centers for Disease Control and Prevention for students in 9th‐12th grades found that 3.2% of students nationally and 1.4% of students in Indiana had initiated sexual intercourse before age 13.^5^ Early sexual onset, however, can typically be defined as having sex prior to an age range between 13 and 15,^5^, ^6^, ^7^ and can lead to poor health outcomes, such as unplanned pregnancy, sexually transmitted infections, and depression.^8^, ^9^, ^10^, ^11^ Adolescents who have their first sexual experience at younger ages have more sexual partners and more unprotected sex.^12^, ^13^, ^14^

### Social ecological model

Social ecological model is used frequently in studies that identify the risk factors related to early sexual onset.^4^, ^15^, ^16^ Developed in the 1970s,^17^ this model has been used by public health workers to promote behavioral changes.^18^ It starts at the internal level and works outward to the systems level or policy level. The framework is a useful approach when investigating a health issue in which influences from different levels all contribute to health behaviors. We examine the influence on abstinence from 3 of the levels, the individual, the interpersonal (family, friends, partners), and environmental (system‐involvement and trauma). Effective interventions are frequently multilevel,^19^ where not just one approach will be successful.

## INDIVIDUAL AND INTERPERSONAL FACTORS

Across a range of populations, research has identified gender, the inability to negotiate safer sex, poor academic performance, and lower sexual health knowledge as individual factors associated with early sexual onset.^4^ However, many of these studies surveyed older adolescents and asked them to reflect back, sometimes several years, introducing recall bias. In a recent meta‐analysis of cohort studies related to early sexual onset, the authors found that adolescent substance use, aggression and conduct disorders, family attachment, school achievement, family living situation, and maternal education were important risk factors associated with early sexual onset.^10^

Attitudes, particularly those related to gender, sexual and relationship behaviors, and family contexts, are considered important to decision‐making around the initiation of sexual behavior.^2^, ^20^ Specifically, beliefs about abstinence and sexual agency (the ability to say “no” or “yes” to sex) contribute to the intention to delay sexual initiation.^20^

### Environmental factors

Experiencing trauma as a youth can have negative lasting consequences throughout the life course.^21^ Multiple studies have demonstrated the relationship between a higher number of adverse childhood experiences (ACEs) and early sexual onset.^22^, ^23^, ^24^ For example, system involvement at the child welfare and juvenile justice level are both identified in the ACEs checklist and have been associated with early sexual onset.^25^, ^26^

### Rural populations

Rural adolescents are particularly at risk for unintended pregnancy, and, nationally, adolescent birth rates among rural populations are higher than those among urban populations.^27^ National data in 2017‐2018 revealed an additional 7.8 births per 1,000 females ages 15‐19 years in rural compared to urban counties.^28^ Similar to national data, the 15 counties with the highest birth rates in Indiana are all rural.^29^

Most studies of early sexual onset in the United States are conducted in urban areas, and the middle school Youth Risk Behavior Surveillance Survey is primarily conducted in urban areas. The 2 longitudinal studies identifying factors related to early sexual were nationally representative and in larger cities.^30^, ^31^ Studies that focus on rural settings are typically performed in low‐ to middle‐income countries.^32^, ^33^, ^34^ One study performed collected data from 225 ninth‐grade students in rural counties in the southern United States from 2006 to 2009. They identified lower religiosity, lower social connectedness, and lower parental monitoring, which were associated with early sexual onset.^35^ Data are lacking for U.S.‐based rural populations, and we need to seek factors across multiple levels of adolescents’ behavior influence.

The primary objective of this study is to identify the intrapersonal, interpersonal, and environmental factors related to early sexual onset and the intention to have or delay sex in the future for youth in rural middle schools at the time when these decisions are being made. A better understanding of the baseline needs of rural middle school students can inform the selection, adaptation, and implementation of sex education programming.

## METHODS

### Study population and procedures

Surveys for this cross‐sectional study were administered to middle school youth in a health class before the implementation of an evidence‐based sex education program in the years 2016‐2019. The larger project was a federally funded teen pregnancy prevention implementation project to Health Care Education and Training, Inc. serving rural students in 21 middle or intermediate schools located in 11 different Southern and Southeastern Indiana counties with high rates of hepatitis C and HIV. Students could be in sixth, seventh, or eighth grades, depending upon when the school chose to offer the programming. The program was implemented by 3 different partner agencies. Participants were nested in schools. All schools in the sample were either rural with larger towns or rural based on the Purdue Rural Indiana Classification System.^36^ This classification was utilized to better capture rurality. Procedures and surveys were approved by the schools. This project was determined to be exempt by the Indiana University Institutional Review Board. Anonymous surveys were collected on paper forms and then entered into a REDCap database. Parental permission was obtained for the program and surveys. All youths in the program were invited to fill out an anonymous survey prior to starting the program, and they were instructed they could choose not to do the survey or stop it at any time.

## MEASURES

The surveys were codeveloped by the implementing organization and external evaluators. Survey measures captured required federal performance measures including age, gender, race/ethnicity, and setting, as well as key goals and objectives related to adolescent pregnancy prevention for the communities they served. Measures of key goals and objectives included previously published measures, some adapted for the age of student or rural contexts (see Ott et al. for a detailed description of measures).^37^ Table 1 has a detailed description of measures. The 2 outcome measures included whether the youth had ever had sex (*Have you ever had sexual intercourse?*) and whether they planned to abstain until the end of high school. Both were yes/no items. The survey included individual items such as age, grade, gender, race, ethnicity, attitudes about abstinence,^38^ and baseline knowledge about sexual topics. Interpersonal items included agency to refuse sex, dating violence items, parent‐child communication scale about sex and relationships.^39^ Environmental items included youth system involvement with child welfare and juvenile justice. Local partners and schools provided feedback and had final approval of the survey.^37^

**TABLE 1:** Details of the survey measures and scales used in bivariate and multivariable models.

Scale name	Number of items (range)	Cronbach's alpha	Answer options	Example item
Positive attitude about abstinence	5 (5‐20)	0.74	4‐point scale from strongly disagree to strongly agree	It is important for me not to have sex before I get married.
Sexual health knowledge score	4	N/A	True/False	You can get pregnant by having sex once without using a condom or other birth control.
Agency for sexual refusal	2 (2‐8)	0.60	4‐point scale from strongly disagree to strongly agree	I feel confident in saying “no” to sex.
Believe that physical violence is ok	1 (1‐4)	N/A	4‐point scale from strongly disagree to strongly agree	Hitting, pushing, kicking, and throwing things at each other is normal in dating relationships.
Parent communication about sex	4 (4‐12)	0.76	3‐point scale never, once or twice, many times	How often have you talked to your parents/caregivers about sex?
Parent communication about relationships	7 (7‐21)	0.82	3‐point scale never, once or twice, many times	How often have you talked to your parents/caregivers about healthy relationships or dating?

### Statistical analyses

Of the 6,799 youth who completed the survey, 6,390 (94.0%) answered yes or no to the question of whether they had ever had sex and 6,572 (96.7%) answered the question regarding the intention to abstain until the end of high school. Those who answered uncomfortable answering the question about sex were excluded from the analysis (n = 409). Because it was not clear why youth chose “uncomfortable,” sensitivity analyses were performed with those who answered “uncomfortable” to the outcome items, with “uncomfortable” recoded first as Yes, then as No, and then as missing. Results of the models run under different recoding were similar, and “uncomfortable” was recoded as missing. We used chi‐square and *t*‐tests to examine bivariate associations between the binary outcomes and sociodemographic, attitude, knowledge, agency, and communication measures. We additionally tested interactions with gender for each of the predictor measures. We found no significant interactions with gender, so both genders were analyzed together. Logistic regression was used to examine the associations between all factors significant at the bivariate level with the outcome of interest. We did not include race in the final multivariable models as this population was mostly White (80.7%), and race was not significant in any of the multivariable models tested. We also removed the dating violence measures due to their impact on the models’ fit. They were not significant in any of the multivariable models. The need for nesting at the partner agency level was investigated for each model using unconditional mixed effects models. We used a threshold of the intraclass correlation (ICC) greater than 0.05. The model for ever had sex (ICC = 0.13) was adjusted for partner agency. All analyses were performed in SPSS Version: 28.0.1.1^40^ and STATA 17.^41^

## RESULTS

The sample consisted of 6,799 adolescents with a mean age of 12.7 (SD = 0.9) years ranging from 10 to 15 years old. The respondents were nearly evenly split between female and male (50.3% and 48.8%, respectively) and 0.9% identified as noncisgender. Overall sample characteristics and bivariate analyses are provided in Table 2. The sample was 80.7% White, 10.5% more than 1 race, 3.0% Native American/Alaskan Native/American Indian, 1.7% other, 1.1% Black or African American, 1.1% unknown, 1.0% Hispanic and/or Latin, and 0.6% Asian or South Asian. A small proportion had a history of child welfare involvement (5.2%) and juvenile justice involvement (4.0%).

**TABLE 2:** Demographic and bivariate analysis by ever had sex and plan to abstain from sex until the end of high school among 7,825 adolescents.

	Total sample N = 6,799	Had sex 328, 5.1%	Never had sex 6,062, 94.9%		Plan to abstain 4,859, 73.9%	Do not plan to abstain 1,713, 26.1%	
Variable	mean (SD) or n, %	mean (SD) or n, %	mean (SD) or n, %	*P*	mean (SD) or n, %	mean (SD) or n, %	*P*
Age, in years, *Range (10‐15)*	12.7 (0.9)	13.4 (0.9)	12.7 (0.9)	^*^	12.6 (0.9)	12.9 (0.9)	^*^
Grade				^*^			^*^
6th	1,667, 24.8%	31, 9.5%	1,559, 26.0%		1,300, 27.0%	295, 17.4%	
7th	3,166, 47.0%	145, 44.5%	2,808, 46.8%		2,256, 46.9%	821, 48.4%	
8th	1,898, 28.2%	150, 46.0%	1,638, 27.3%		1,254, 26.1%	582, 34.3%	
Gender				^*^			^*^
Female	3,406, 50.3%	126, 38.7%	3,118, 51.5%		2,676, 55.2%	640, 37.5%	
Male	3,310, 48.8%	191, 58.6%	2,891, 47.8%		2,138, 44.1%	1,045, 61.2%	
Other	62, 0.9%	9, 2.8%	42, 0.7%		38, 0.8%	22, 1.3%	
Race				^*^			^*^
White or Caucasian	5,485, 80.7%	228, 69.5%	4,966, 81.9%		3,979, 81.9%	1,335, 77.9%	
Native American/Alaska Native/American Indian	204, 3.0%	19, 5.8%	165, 2.7%		133, 2.7%	65, 3.8%	
Native Hawaiian/Other Pacific Islander	23, 0.3%	7, 2.1%	14, 0.2%		15, 0.3%	7, 0.4%	
Black or African American	72, 1.1%	8, 2.4%	61, 1.0%		45, 0.9%	22, 1.3%	
Asian or South Asian	41, 0.6%	1, 0.3%	37, 0.6%		30, 0.6%	6, 0.3%	
Other	115, 1.7%	7, 2.1%	97, 1.6%		80, 1.6%	40, 1.9%	
Latin	66, 1.0%	6, 1.8%	54, 0.9%		41, 0.8%	27, 1.3%	
More than 1 race	716, 10.5%	48, 14.6%	602, 89.9%		481, 9.9%	309, 15.0%	
Unknown/Not reported	77, 1.1%	4, 1.2%	66, 1.1%		55, 1.1%	15, 0.7%	
Positive attitude about abstinence *5 items, Range 5‐20 α = 0.74*	15.7 (3.0)	11.3 (2.9)	15.8 (3.0)	^*^	16.4 (2.7)	13.0 (3.1)	^*^
Sex knowledge *4 items*, *Range 0‐4*	3.4 (0.9)	3.3 (0.9)	3.4 (0.9)	^*^	3.4 (0.9)	3.2 (0.9)	^*^
Agency for sexual refusal *2 items, Range 2‐8, α = 0.60*	6.7 (1.4)	5.8 (1.6)	6.9 (1.3)	^*^	7.0 (1.2)	6.1 (1.5)	^*^
Believe physical violence is ok *1 item, Range 1‐4*	1.4 (0.7)	1.5 (0.9)	1.4 (0.6)	^*^	1.4 (0.6)	1.5 (0.7)	^*^
Believe checking partners’ cell is ok *1 item, Range 1‐4*	2.0 (0.9)	2.3 (1.1)	2.0 (0.9)	^*^	1.9 (0.8)	2.1 (1.0)	^*^
Parent communication on sex *4 items, Range 4‐12, α = 0.76*	5.9 (2.0)	7.6 (2.6)	5.7 (1.8)	^*^	5.7 (1.8)	6.3 (2.3)	^*^
Parent communication on relationships *7 items, Range 7‐21, α = 0.82*	11.7 (3.5)	13.5 (4.1)	11.6 (3.4)	^*^	11.6 (3.4)	12.0 (3.7)	^*^
History of child welfare				^*^			^*^
Yes	354, 5.2%	57, 17.5%	258, 4.3%		206, 4.3%	141, 8.4%	
No	6,368, 94.7%	268, 82.5%	5,746, 95.7%		4,615, 95.7%	1,546, 91.6%	
History of juvenile justice				^*^			^*^
Yes	267, 4.0%	85, 25.9%	129, 2.1%		93, 1.9%	159, 9.4%	
No	6,488, 96.0%	243, 74.1%	5,901, 97.7%		4,743, 98.1%	1,541, 90.6%	
County income (in thousands)	57.6 (6.7)	56.6 (7.2)	57.6 (6.7)	^*^	57.9 (6.7)	56.9 (6.8)	^*^

* *P*<.05.

For the first outcome, a total of 328 (5.1%) adolescents reported that they had ever had sex, 6,062 (94.4%) reported they did not have sex, and 409 answered “I'm uncomfortable answering this question” and were excluded from the analyses. Multi‐level logistic regression adjusted for partner agency and all other variables in the model (Table 3) revealed that ever having sex was associated with being older (OR = 1.17, 95% CI: 1.04‐1.32), less positive attitudes toward abstinence (OR = 0.66, 95% CI: 0.63‐0.70), less ability of agency for sexual refusal (OR = 0.83, 95% CI: 0.74‐0.92), having a history of child welfare involvement (OR = 2.57, 95% CI: 1.64‐4.02), having a history of juvenile justice involvement (OR = 4.50, 95% CI: 2.87‐7.09), and more frequent parent communication about sex (OR = 1.30, 95% CI: 1.16‐1.45).

**TABLE 3:** Logistic regression analysis of the associations between risk factors and ever having sex among 6,390 adolescents.

Factors^a^	n or range^b^	OR1 (95% CI)^c^	OR2 (95% CI)^d^
Age in years	10‐15	1.49 (1.31, 1.70)	1.17 (1.04, 1.32)
Gender			
Male	3,039	1.62 (1.29, 2.04)	0.74 (0.54, 1.03)
Female	3,279	ref.	ref.
Positive attitude toward abstinence	5‐20	0.61 (0.58, 0.63)	0.66 (0.63, 0.70)
Higher sexual knowledge	0‐4	0.87 (0.78, 0.99)	1.0 (0.80, 1.17)
Better agency for sexual refusal	2‐8	0.62 (0.58, 0.67)	0.83 (0.74, 0.92)
More parent communication about sex	4‐12	1.45 (1.38, 1.52)	1.30 (1.16, 1.45)
More parent communication about relationships	7‐21	1.15 (1.11, 1.18)	0.97 (0.91, 1.04)
History of child welfare involvement			
Yes	303	4.52 (3.30, 6.18)	2.57 (1.64, 4.02)
No	6,015	ref.	ref.
History of juvenile involvement			
Yes	212	15.47 (11.39, 21.03)	4.50 (2.87, 7.09)
No	6,106	ref.	ref.
Median county income (in thousands)	45.8‐65.8	0.97 (0.95, 0.99)	1.00 (0.98, 1.02)

^a^ All factors were analyzed as continuous variables except gender, history of child welfare involvement, and history of juvenile involvement.

^b^ n for categorical variables and range for continuous variables.

^c^ Adjusted for partner agency.

^d^ Adjusted for partner agency and all other factors listed in Table 3.

For the second outcome, 4,859 (73.9%) adolescents planned to abstain until the end of high school. Controlling for all variables in the model, logistic regression indicated that adolescents who planned to abstain from sex until the end of high school (Table 4) were younger (OR = 0.80, 95% CI: 0.73‐0.86), had more positive attitudes toward abstinence (OR = 1.48, 95% CI: 1.43‐1.52), higher sexual knowledge scores (OR = 1.14, 95% CI: 1.04‐1.24), better agency for sexual refusal (OR = 1.18, 95% CI: 1.11‐1.30), and more communication with parents about relationships (OR = 1.04, 95% CI: 1.01‐1.08). Not planning to abstain until the end of high school was associated with more parent communication about sex (OR = 0.85, 95% CI: 0.80‐0.91), a history of child welfare involvement (OR = 0.71, 95% CI: 0.52‐0.97), and a history of juvenile justice involvement (OR = 0.62, 95% CI: 0.43‐0.90).

**TABLE 4:** Logistic regression analysis of the associations between risk factors and planning to abstain from sex until the end of high school among 6,572 adolescents.

Factors^a^	n or range^b^	Crude (95% CI)	Adjusted (95% CI)^c^
Age in years	10‐15	0.68 (0.64, 0.72)	0.80 (0.73, 0.86)
Gender			
Male	3,150	0.49 (0.44, 0.55)	0.72 (0.62, 0.84)
Female	3,372	ref.	ref.
Positive attitude toward abstinence	5‐20	1.58 (1.53, 1.62)	1.48 (1.43, 1.52)
Higher sexual knowledge	0‐4	1.18 (1.11, 1.25)	1.14 (1.04, 1.24)
Better agency for sexual refusal	2‐8	1.60 (1.53, 1.67)	1.18 (1.11, 1.30)
More parent communication about sex	4‐12	0.85 (0.83, 0.87)	0.85 (0.80, 0.91)
More parent communication about relationships	7‐21	0.97 (0.96, 0.99)	1.04 (1.01, 1.08)
History of child welfare involvement			
Yes	343	0.49 (0.39, 0.61)	0.71 (0.52, 0.97)
No	6,179	ref.	ref.
History of juvenile involvement			
Yes	264	0.19 (0.15, 0.25)	0.62 (0.43, 0.90)
No	6,258	ref.	ref.
Median county income (in thousands)	45.79‐65.84	1.00 (1.00, 1.00)	1.01 (0.99, 1.02)

^a^ All factors were analyzed as continuous variables except gender, history of child welfare involvement, and history of juvenile involvement.

^b^ n for categorical variables and range for continuous variables.

^c^ Adjusted for all factors listed in Table 4.

## DISCUSSION

Middle school youth from rural counties had a 5.1% rate of early sexual onset before age 16. While we are unable to compare this to national samples collected in high school, our sample was younger, comprised of mostly 12‐ and 13‐year‐olds. However, 23.4% of our sample's 15‐year‐olds had reported sex compared to earlier Add Health data waves 1, 3, and 4, where 16.6% reported sex before age 16.^7^ Our data were collected from youth closer to the event, potentially increasing accuracy in the measurements of attitudes, knowledge, and behaviors, and informing sexuality education programs for middle school youth.

When we investigated the individual factors, older age was associated with ever having sex and the intention to have sex in the future indicating a need for early interventions. Integrating these programs is particularly important, because there have been declines in formal sexuality education in rural areas.^42^ Our data support the need for additional strategic interventions for rural youth in middle schools.

Gender was associated with the intention to delay in the future, but not associated with ever having sex. While males were more likely to intend to have sex in the future, there was no difference in the likelihood of already having sex. More masculine values have been associated with both outcomes in previous literature,^43^ pointing to early adolescence as an important time for prevention efforts focused on young men.

Those with more positive attitudes around the idea of abstinence were less likely to have had sex and more likely to intend to delay sex. These attitudes may be important modifiable targets for sex education interventions for early adolescents. However, for older adolescents, abstinence attitudes have not been shown to be sufficient to prevent unprotected sex. Masters et al. found that youth who do not consider abstinence and sexual activity as opposing constructs may prevent sexual activity, but those intentions may make them less equipped to use contraception or condoms at their first sexual intercourse.^44^ Thus, it is important to provide early abstinence teaching alongside the other components of comprehensive sex education, such as condom and contraception use.

For interpersonal factors, our data suggest that building skills around sexual agency may be effective in delaying sexual onset. Higher agency for sexual refusal was associated with never having sex and the intention to delay sex in the future. Agency and refusal skills have been effective components of sex education in middle schools, but the study was in urban areas.^45^ Our research supports the need of agency and refusal skills before high school in rural communities that are also high‐risk.

Finally, within the environmental dimension of the social ecological model, we identified that the trauma of being involved in child welfare and juvenile justice impacted both past behavior and future intention regarding sex. Our data highlight the need for trauma‐informed approaches to comprehensive sex education. Juvenile justice involvement in our study was one of the highest predictors of sexual onset.

This study fills gaps in the research about the sexual behaviors and intentions of middle school youth in rural settings of a Midwest state in the United States. However, we are limited by its cross‐sectional design. Because rates of sexual behavior were low, we additionally examined intention to abstain as an intermediate outcome. Although we are not able to know if intentions held over time, we captured the attitudes and behaviors of youth when these decisions are being made. We were also constrained by how much we could ask the youth, as this was implemented in schools, as we were only allotted a small amount of class time to survey youth and needed to get administration as well as IRB approval.

Overall, our study identifies important risk factors and provides modifiable factors to inform the sparse research to date, on pregnancy prevention with younger samples from rural populations in the United States. Our research supports the recommendations by the American Academy of Pediatrics statement on the importance of access to comprehensive and trauma‐informed sex education.^46^ Our focus on rural areas is in response to the high teen birth rates in rural counties compared to urban counties.^27^, ^28^, ^29^ Our findings highlight the importance of comprehensive, trauma‐informed sex education for youth before high school, the need to engage parents early, the benefit of talking about sexual agency and consent, and the importance of attitudes and behaviors associated with abstinence.

## CONFLICT OF INTEREST STATEMENT

Dr. Ott discloses that her spouse is an employee of Eli Lilly, Inc., and that together they are joint small stockholders. Eli Lilly does not produce pregnancy prevention products. The remaining authors have no conflicts of interest to declare.
